# Outcomes of Post-Keratoplasty Microbial Keratitis: A 16-Year Analysis

**DOI:** 10.3390/jcm14093165

**Published:** 2025-05-03

**Authors:** Joanna Przybek-Skrzypecka, Małgorzata Ryk-Adamska, Janusz Skrzypecki, Justyna Izdebska, Monika Udziela, Joanna Major, Jacek P. Szaflik

**Affiliations:** 1 Department of Ophthalmology, Medical University of Warsaw, Sierakowskiego 13, 01-756 Warsaw, Poland; malgorzata.ryk-adamska@wum.edu.pl (M.R.-A.); justyna.izdebska@wum.edu.pl (J.I.); monika.udziela@wum.edu.pl (M.U.); jacek.szaflik@wum.edu.pl (J.P.S.); 2 SPKSO Ophthalmic University Hospital, 03-709 Warszawa, Poland; jmajor@interia.pl; 3 Department of Experimental Physiology and Pathophysiology, Medical University of Warsaw, 02-091 Warszawa, Poland; janusz.skrzypecki@wum.edu.pl

**Keywords:** keratitis, corneal ulcers, corneal transplant, visual acuity, immunosuppression

## Abstract

**Purpose:** To determine the incidence, risk factors (including systemic immunosuppression), and outcomes of microbial keratitis in corneal transplants over a 16-year observation period at a tertiary referral hospital in Poland. **Methods:** This retrospective cohort study included 125 episodes of infectious keratitis in 117 patients who underwent corneal transplantation between 2008 and 2023 at the Department of Ophthalmology, Medical University of Warsaw, Poland. The data collected included demographics, indications for transplantation, infection rates, risk factors, best-corrected visual acuity (BCVA) at presentation and discharge, changes in visual acuity, and treatments received prior to hospital admission. Clinical signs, symptoms, diagnostic tests, and management strategies were also reviewed. Additionally, the outcomes of surgical interventions, such as therapeutic corneal transplantation and evisceration, were examined. **Results**: Among the 2869 corneal transplants performed over the 16-year period, the incidence of post-keratoplasty microbial keratitis (PKMK) was 4.35%. The most common indication for transplantation in affected patients was an active infection unresponsive to medical therapy (n = 62, 52%). One-third of PKMK cases occurred in patients with repeat transplants. Median visual acuity prior to infection was 1.6 logMAR, worsening to 2.3 logMAR at presentation. Following treatment, visual acuity improved to a median of 1.9 logMAR at discharge, with no significant improvement by the one-year follow-up. At that time, 75.1% of patients remained legally blind (BCVA ≤ 20/200); 21% recovered to pre-infection visual levels, while 46% experienced additional visual loss due to PKMK. Multivariate regression identified corneal perforation and systemic immunosuppression as independent predictors of poorer visual outcomes (*p* < 0.001 and *p* = 0.03, respectively. **Conclusions**: Microbial keratitis in corneal grafts is associated with poor long-term visual outcomes. At one year post-infection, the median BCVA was 1.9 logMAR, with 75.1% of patients remaining legally blind. Nearly half of the cohort experienced additional visual loss compared to their pre-infection status, underscoring the severity of PKMK and the need for vigilant postoperative care.

## 1. Introduction

Corneal transplantation enhances vision and improves health-related quality of life for its recipients [1]. Both patients and healthcare providers strive to preserve the clarity of the transplanted cornea for as long as possible. However, several well-documented complications can threaten its success, including graft failure, graft rejection, secondary glaucoma, and post-keratoplasty microbial keratitis (PKMK) [2]. Among these, PKMK poses the most significant risk, as it not only reduces corneal transparency but may also lead to complete eyeball loss due to uncontrolled infection [3]. Furthermore, PKMK results in corneal decompensation in 60–70% of cases, often requiring repeat keratoplasty [4]. Given the global shortage of donor corneas—13 million people are currently on the waiting list—as well as the long waiting times and potential complications associated with repeat transplantation, every effort must be made to prevent infection and identify factors that influence final visual outcomes [5].

The reported incidence of keratitis after penetrating keratoplasty ranges from 0.02% to 18.8%, varying by country and significantly lower in posterior lamellar transplants [6,7]. Several risk factors for PKMK have been identified, including persistent epithelial defects, dry eye syndrome, prior corneal transplantation due to infectious keratitis, fungal etiology, topical immunosuppression, ulcer size > 60 mm, and a history of topical or surgical glaucoma treatment [8,9]. However, the real-world impact of PKMK on vision loss and its underlying contributing factors remains poorly understood. Given the significant burden of intensive anti-infective treatments and frequent hospitalizations, it is essential to evaluate whether these interventions achieve their intended goal: preserving or restoring vision. From the patient’s perspective, understanding the potential visual outcomes of such intensive therapy is critical. Therefore, this study aims to evaluate actual vision loss by comparing best-corrected visual acuity (BCVA) at three key timepoints: the last visit before infection onset, hospital discharge following PKMK, and the one-year follow-up visit.

The primary objective of this study was to determine the incidence, predisposing factors, and clinical outcomes of post-keratoplasty microbial keratitis, including visual acuity changes and the need for surgical intervention, in a cohort of patients treated over a 16-year period at the Department of Ophthalmology, Medical University of Warsaw. All patients had a minimum follow-up of one year. The secondary objective was to assess the impact of systemic immunosuppression on the clinical course and visual outcomes of PKMK.

## 2. Methods

A retrospective analysis was conducted on 117 adults diagnosed with severe microbial keratitis who were either hospitalized or treated as outpatients at the Department of Ophthalmology, Medical University of Warsaw, between 2008 and 2023. The total number of corneal transplants performed at the same tertiary eye center during this period was also reviewed. This study received approval from the Institutional Review Board of the Medical University of Warsaw (approval number: 286/2024).

“Microbial keratitis” was defined as either microbial growth in corneal cultures or clinical improvement following antimicrobial treatment. This study adhered to the principles of the Declaration of Helsinki.

The cohort was divided into subgroups based on the type of keratoplasty performed:

- Full-thickness corneal transplant: Penetrating keratoplasty (PK);
- Posterior lamellar keratoplasty: Descemet stripping automated endothelial keratoplasty (DSAEK);
- Anterior lamellar keratoplasty: Deep anterior lamellar keratoplasty (DALK).

Of note, Descemet membrane endothelial keratoplasty (DMEK) was not performed in our hospital during the study period.

### 2.1. Data Collection

Data were collected on the following variables: patient demographics (age and sex), duration of hospitalization, presence of active uveitis, past ocular history, and systemic comorbidities. Information regarding the indications for keratoplasty and the number of prior transplants was also recorded. Additionally, potential risk factors for microbial keratitis were assessed, including contact lens use, ocular trauma, dry eye syndrome, broken or loose sutures, and a history of recurrent corneal erosions.

Additional variables analyzed included: prior treatments; time from symptom onset to ophthalmologic evaluation; corneal status prior to ulcer formation (categorized as decompensated or not); presence of corneal perforation; lens status; history of glaucoma (medical or surgical); clinical manifestation of the infection (monofocal vs. multifocal); location of the infection (central, defined as within a 5 mm diameter; peripheral or mid-peripheral (diameter within 5–8 mm); or whole corneal involvement); and general health status, with particular attention to autoimmune diseases that could impair immunocompetence (e.g., diabetes mellitus, atopic dermatitis, psoriasis, rheumatoid arthritis, and systemic lupus erythematosus).

Empirical therapy was categorized into:

(a) Topical therapy: antibacterial treatment included either monotherapy (using a fluoroquinolone, aminoglycoside, or chloramphenicol) or combination therapy (gentamicin plus vancomycin, or a fluoroquinolone combined with an aminoglycoside). Additional topical therapies comprised antifungal, antiviral, anti-amoebic agents, and corticosteroids.
(b) Systemic therapy: immunosuppression.

Special attention was given to general immunosuppression (IMT). According to the local guidelines for high-risk corneal transplants, mycophenolate mofetil (1 g twice daily, orally) is the standard immunosuppressive therapy. Other immunosuppressive agents included glucocorticoids, azathioprine, methotrexate, cyclosporine, and tacrolimus. IMT was initiated on the day of the keratoplasty procedure in patients undergoing high-risk corneal transplantation. High-risk grafts were defined by the presence of one or more of the following features: neovascularization involving at least two limbal quadrants, repeated grafting, corneal decompensation, or surgery performed during an active infection phase, all of which were associated with an increased risk of graft failure. An additional indication for IMT was the use of large donor grafts (diameter greater than 9.5 mm). Treatment was discontinued according to the local protocol, typically one year after initiation, or earlier in the event of severe adverse effects. Timing and dosages of IMT cessation were also recorded.

Visual acuity outcomes were evaluated as best-corrected distance visual acuity (BCVA) at four timepoints: initial presentation, hospital discharge, one year after the infectious episode, and at the last visit prior to the onset of corneal ulceration (within a maximum of three months). Visual acuity was measured using the Logarithm of the Minimum Angle of Resolution (logMAR) scale, in accordance with the World Health Organization (WHO) classification. Extremely low visual acuity levels were also recorded, assigning standardized logMAR values: hand movements (2.3 logMAR), light perception (2.7 logMAR), and no light perception (3.0 logMAR). Lens status was assessed to exclude cataract-related bias in VA measurements. Legal blindness was defined according to the WHO criteria as a logMAR score ≥ 1.0 (equivalent to 20/200 or 5/50 Snellen acuity). Additionally, this study recorded the incidence of therapeutic keratoplasty, amniotic membrane transplantation, and evisceration, as documented in the medical records.

### 2.2. Statistical Methods

Analysis was carried out using R statistical software, version 4.4.2. the data are presented with n (% of group) for nominal variables and as mean ± standard deviation (SD) or median (Q1; Q3) for continuous variables, depending on distribution normality. Nominal variables were presented with absolute and relative number of observations. Shapiro–Wilk test, skewness, and kurtosis were used to verify distribution normality. Levene’s test was employed to verify variance homogeneity. Comparisons of numeric variables between groups were performed with the t-Student test or Mann–Whitney U test, as appropriate. Nominal variables were compared between groups with Pearson’s chi-squared test or Fisher’s exact test, as appropriate. Two-step linear regression analysis was employed to identify factors associated with a relative change in BCVA. Cut-off at the level of *p* = 0.157 was used for the pre-selection of variables into a multivariate step. Furthermore, the stepwise selection algorithm was run for the final selection. Multivariate model fit was verified with R2 and adjusted R2. All calculations assumed significance if *p* < 0.05.

## 3. Results

### 3.1. Demographics

The analysis included 125 episodes of infectious keratitis in 117 patients who had previously undergone corneal transplantation. These cases represented 4.35% of the 2869 corneal transplants performed between 2008 and 2023 at the Department of Ophthalmology, Medical University of Warsaw, a tertiary referral eye center. The incidence of infectious keratitis varied by type of transplant: 5.9% following penetrating keratoplasty, 0.37% following Descemet stripping automated endothelial keratoplasty, and 8.8% following deep anterior lamellar keratoplasty.

Females comprised the majority of the cohort (64%), with a median age of 61 years. Patients who underwent DSAEK were older, with a mean age of 76 years. This study included 112 episodes of PKMK following PK, 3 episodes following DSAEK, and 10 episodes following DALK. No significant differences were observed in the prevalence of systemic autoimmune diseases among patients undergoing different types of corneal transplantation. The majority of patients (114 cases, 93%) required hospital admission. Detailed demographic characteristics are presented in Table 1.

The clinical history of corneal transplants is summarized in Table 2. A history of repeated corneal transplantation was noted in nearly one-third of the cohort (40 patients, 32%). Among these, most patients had undergone two transplants (29 patients), followed by three transplants (8 patients), four transplants (2 patients), and five transplants in one patient. In this group, the median interval from the first corneal transplant to the onset of microbial keratitis was 76 months (interquartile range [IQR]: 34.25–143.75 months).

The most common indication for corneal transplantation was active infection, including 16 cases of perforated microbial keratitis, requiring therapeutic transplants in 62 eyes. Other indications included bullous keratopathy (22 cases), post-infectious corneal scars (13 cases), and keratoconus (12 cases).

The median time from the last corneal transplant to the onset of microbial keratitis was 17 months (IQR: 6.0–40.0) for the entire cohort and for patients who underwent PK patients. In contrast, DSAEK patients developed PKMK earlier, with a median time of 7 months.

### 3.2. Risk Factors and Clinical Characteristic

Contact lens use and a history of trauma were each reported in a small number of patients (2 and 3 patients, respectively). More than half of the cohort (54.7%) was receiving topical antiglaucoma therapy, and 23.9% of eyes had undergone at least one glaucoma surgery. Prior to the onset of PKMK, 54.7% of the corneal grafts were decompensated.

Additional risk factors for PKMK, categorized by transplant type, are presented in Table 3. Based on the use of systemic immunosuppression, the cohort was further divided into two groups: patients receiving immunosuppressive therapy and those not receiving IMT, corresponding to high-risk and standard-risk corneal transplants, respectively (Table 4). Notably, best-corrected distance visual acuity was consistently better in the IMT group at all stages of observation, with statistically significant differences observed both at the last visit prior to PKMK onset and at the time of PKMK diagnosis.

### 3.3. Visual Acuity

The median visual acuity at admission was 2.3 logMAR (Q1:Q3 = 1.5–2.3) for the entire cohort decreasing from the median 1.6 logMAR (Q1:Q3= 0.88–2.3) obtained at the visit preceding the infection. At the one-year follow-up visit, median BCVA improved to median 1.9 logMAR (Q1:Q3 = 0.9–2.3) with a discrepancy observed between the transplant’s types shown in Table 5.

Outcomes of PKMK were evaluated one year after the onset of infection. The primary outcome was defined as the change in logMAR visual acuity between the visit immediately preceding the PKMK episode and the one-year follow-up visit. The relative change in BCVA ranged from −1.50 to 2.70 logMAR units, with a median change of 0.00. In real-world outcomes, 37 patients (32.7% of the cohort) demonstrated an improvement in BCVA, 24 patients (21.2%) had stable BCVA, and 52 patients (46.1%) experienced a deterioration in BCVA following PKMK treatment.

A parallel analysis was performed on patients who underwent corneal scraping for microbiological culture (n = 37), divided into scrape-positive and scrape-negative groups. The only statistically significant difference between the groups was observed at the time of discharge, with a median BCVA of 2.3 logMAR in the scrape-positive group compared to 1.55 logMAR in the scrape-negative group (*p* = 0.04). BCVA at both the time of diagnosis and at the one-year follow-up did not significantly differ between the groups, with a median of 2.3 logMAR at each timepoint.

Lens status did not significantly influence visual acuity outcomes, as indicated by the following distribution: 26 patients with a transparent lens, 13 with cataracts, 65 pseudophakic patients, and 11 aphakic patients. A trend was observed for therapeutic transplants to improve BCVA (*p* = 0.067).

**Figure 1:**
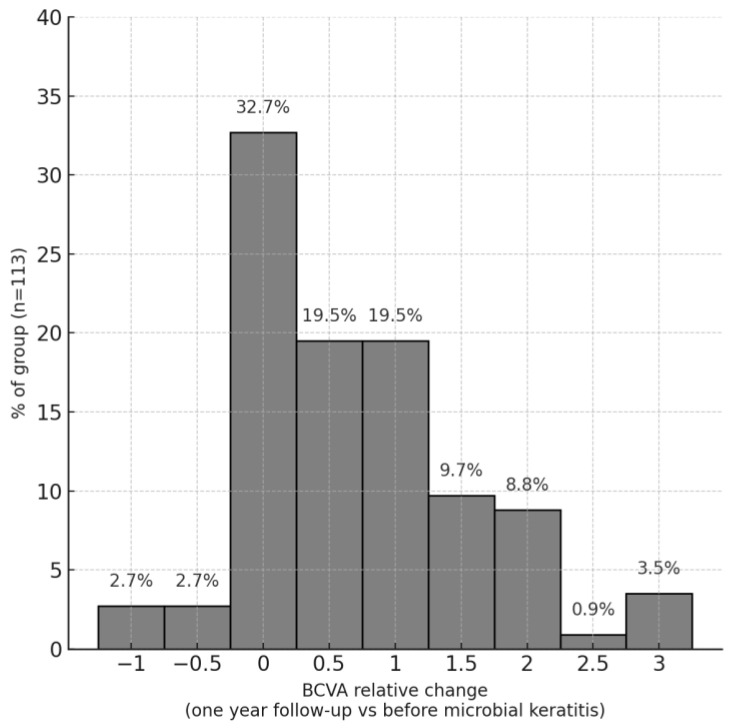
Histogram presenting the distribution of relative change in BCVA (best-corrected visual acuity) defined as the difference between BCVA at year one follow-up visit, and BCVA at the visit preceding microbial keratitis onset, excluding eviscerated eyeballs.

Figure 1 illustrates the distribution of relative changes in BCVA. Eviscerated eyes (eight eyes, 6.4% of the cohort) and patients lost to follow-up after hospital discharge were excluded from this analysis.

**Figure 2:**
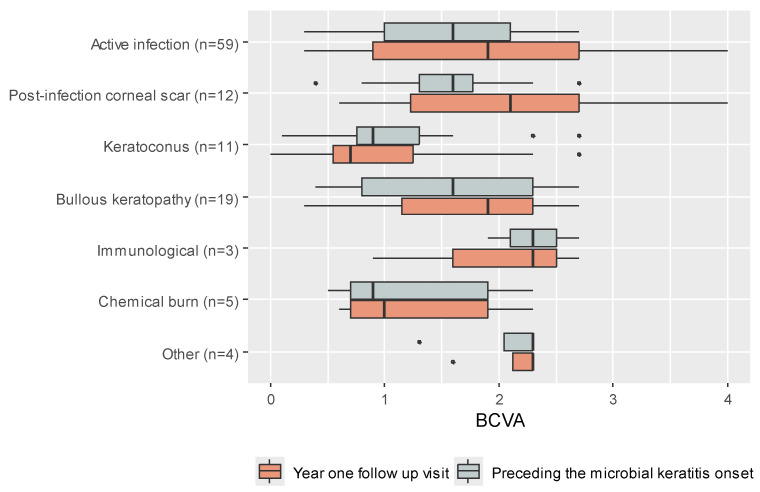
Boxplot presenting the distribution of BCVA (best-corrected visual acuity) preceding microbial keratitis and at the one-year follow-up visit, by indication (dot indicating single patients outside the 95% confidence interval).

**Figure 3:**
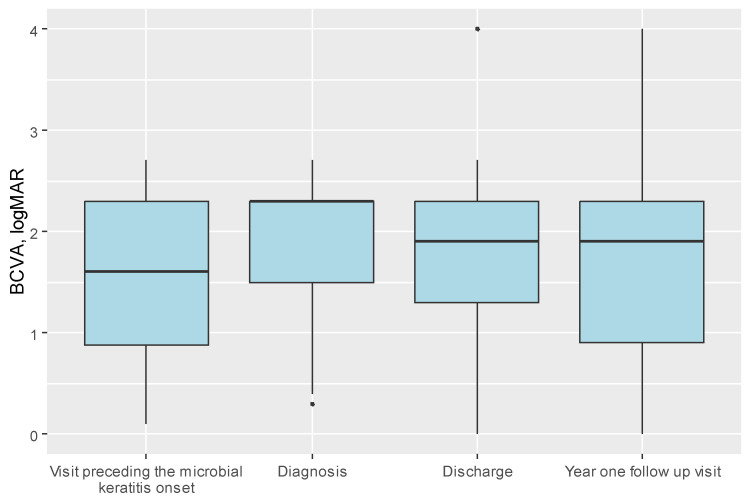
Boxplot presenting the distribution of BCVA before microbial keratitis onset, at diagnosis, at discharge, and at the one-year follow-up visit (*bold line indicates median*).

Figure 2 shows BCVA at two timepoints, divided into subgroups based on the primary transplant indications: (a) prior to the onset of PKMK and (b) one year following PKMK. Figure 3 presents the BCVA at four timepoints for the entire cohort.

In the final stage of our analysis, we examined the management of post-keratoplasty microbial keratitis in two patient groups: those with a history of a single keratoplasty and those who had undergone multiple transplants (n = 40 cases) prior to developing PKMK. We found no statistical difference between the clinical and surgical management of these groups. Table 6 summarizes the various treatment modalities utilized in each group.

Next, univariate and multivariate analyses were performed to assess factors associated with the relative change in best-corrected distance visual acuity. The primary outcome was defined as an improvement of at least one line in BCDA between the visit immediately preceding the infection and the one-year follow-up visit. Table 7 summarizes all variables included in the analysis.

In the univariate analysis, female sex and keratoconus (as the indication for transplantation, compared to active infection) were identified as statistically significant positive predictors of BCVA improvement (*p* = 0.041 and *p* = 0.047, respectively). In contrast, a history of recurrent corneal infections and the presence of corneal perforation at the time of PKMK diagnosis were identified as negative predictors of visual improvement (*p* = 0.04 and *p* = 0.005, respectively). Notably, initial BCVA did not correlate with the final visual outcome.

A multivariate linear regression model was constructed to assess the association between analyzed variables and the change in BCVA, adjusting for dependencies among variables. Time from the first transplant to the onset of microbial keratitis was excluded from the model due to insufficient data. Following the variable selection procedures, the final multivariate model included: transplant type, presence of perforation, and use of local immunosuppression at the time of infection. The analysis demonstrated that undergoing DALK was associated with a 0.65 higher change in BCVA compared to PK transplant, ß = 0.65 CI95 [0.09; 1.21], *p* = 0.024. Perforation at the time of infection was associated with a 0.77 greater change in BCVA (ß = 0.77 CI95 [0.36; 1.17], *p* < 0.001). Additionally, the use of local immunosuppression during infection was associated with a 0.45 greater change in BCDVA (ß = 0.45; 95% CI [0.16, 0.74]; *p* = 0.003) (Table 5). A trend was also observed suggesting that therapeutic keratoplasty may improve final visual acuity, although this did not reach statistical significance.

## 4. Discussion

In this study, we observed a 4.35% incidence of PKMK among 2869 corneal transplants performed over a 16-year period at our tertiary referral center. The incidence of PKMK was 5.9% following PK, 0.37% following DSAEK, and 8.8% following DALK. The majority of corneal ulcers occurred in full-thickness corneal transplants (112 cases, 90%), followed by DALK (10 cases, 8%) and DSAEK (3 cases, 2%), corresponding to a 0.37% rate of PKMK among all posterior lamellar grafts performed. PKMK was most frequently diagnosed in grafts performed for active corneal infection (52% of cases). Overall, the median BCVA decreased from logMAR 1.6 prior to infection to logMAR 1.9 one year after infection. However, BCVA improved relative to the time of PKMK diagnosis, where the median logMAR was 2.3. Perforation and the need for systemic immunosuppression were identified as the most-significant negative predictors of visual outcomes [10].

The literature reports a wide range of PKMK rates, from 0.02 to 0.9% in posterior lamellar grafts, and from 0.4% to 18.8% in cohorts, including penetrating or mixed keratoplasties [11,12]. However, most studies focused on the risk of infection after penetrating keratoplasty report rates comparable to our findings, typically ranging between 4 and 6%. A model study by Wagoner et al. reported a PKMK incidence of 4.9% [13]. One of the largest European studies, conducted by Griffin et al., demonstrated a PKMK rate of 4.77% per graft rate in the United Kingdom [14]. In contrast to many previous studies, we present a substantially larger cohort of patients with PKMK (n = 117) derived from a large database of corneal transplants performed over a 16-year period of time at our tertiary center. The low incidence of infections in posterior lamellar grafts at our center may be partially attributed to the relatively small, although increasing, proportion of DSAEK/DMEK procedures (28.6%, n = 821). For comparison, a large American study with a similar number of transplants (n = 2098) reported a 60/40 distribution between penetrating and posterior lamellar transplants, with an ongoing shift favoring lamellar techniques [10]. Nevertheless, our observed PKMK rate of 0.37% among DSAEK transplants is consistent with the rates reported in other international studies.

Well-established risk factors for PKMK include persistent epithelial defects, broken or loose sutures, prior fungal infection, corneal perforation, corneal sensory dysfunction, glaucoma, and the use of topical glucocorticosteroids [15,16]. While the role of topical immunosuppression in increasing the risk of infection is well-documented, the impact of systemic immunosuppression remains less clearly understood. Glucocorticosteroids and other systemic immunosuppressive agents promote immunologic tolerance, but may also increase susceptibility to corneal infections [17]. Systemic immunosuppression is considered the gold standard for preventing allograft rejection during the first six months following keratoplasty [18], but it is typically reserved for patients undergoing high-risk corneal transplantation [19].

In high-risk corneal transplants, the balance between immune privilege and graft clarity remains poorly defined. Several factors have been investigated to better understand this balance, including the role of anti-human leucocyte antigen (HLA) antibodies [20]. It is well-established that preoperative corticosteroid use increases the risk of graft failure. Similarly, based on immunological mechanisms, it can be hypothesized that systemic immunosuppression may contribute to an increased risk of post-keratoplasty infections and impair their treatment, although the current evidence remains limited. To our knowledge, this is the first study to identify systemic immunosuppression as a negative predictor of favorable outcomes in PKMK. Importantly, BCVA in high-risk transplant recipients (who were receiving systemic immunosuppression) was better prior to the onset of PKMK, indicating that the observed association is unlikely to be confounded by a worse pre-infection visual status.

In our cohort, the primary indication for keratoplasty was aggressive keratitis, affecting more than half of the patients (52%), followed by bullous keratopathy, post-infectious corneal scarring, and keratoconus. Sharma et al. reported that therapeutic grafts generally have a poorer prognosis for graft survival compared to optical grafts [21]. Therefore, the unfavorable visual outcomes observed in our study may be related to the high proportion of infections diagnosed in patients with previous therapeutic keratoplasties. Our findings are consistent with the general trends demonstrating that keratoconus is the most favorable indication for keratoplasty in terms of visual outcomes [22]. In the study by Wagoner et al., keratoconus as an indication for keratoplasty was associated with an 84% graft survival rate following PKMK over a four-year follow-up period [13]. Veugen et al. reported significantly worse graft survival outcomes in therapeutic keratoplasties performed for infectious keratitis with two-year survival rates of 22% for Acanthamoeba keratitis, 70% for viral keratitis, and 86% for bacterial keratitis [23]. Fungal keratitis was also associated with poor prognosis, including higher rates of endophthalmitis and evisceration [24]. Additionally, Chatterjee et al. highlighted that infection recurrence withing therapeutic grafts reached 27.5% for fungal infections [25]. The potentially high rate of fungal infections may partly explain the unfavorable outcomes observed in our cohort. Given the limited availability of the corneal culture results in our study, it can be hypothesized that poor prognosis factors, such as fungal infections, were relatively common—particularly considering the relatively high rate of evisceration. Therefore, it is crucial to emphasize the importance of performing corneal scraping and microbiological analysis, including susceptibility testing, to optimize empirical treatment and improve clinical outcomes.

Visual outcomes are generally poor in cases of microbial keratitis, and even more so in post-keratoplasty microbial keratitis. However, relatively few studies have specifically focused on visual outcomes in this context. Among them, Moon et al. reported a similar median post-PMKM visual acuity of 1.85 logMAR ± 0.91, although their sample size was limited to 19 patients [26]. Tixier et al. found that 63% of PKMK patients ended up with a VA of less than 20/200 despite optimal medical and surgical management. In our study, 75.1% of patients were ultimately classified as legally blind (VA < 20/200). The discrepancy between our results and those of Tixier may be attributed to the significantly larger cohort size in our study (117 patients vs. 18 patients in Tixier’s study), as well as the high proportion of high-risk therapeutic keratoplasties (52% of our cohort) and the high incidence of graft decompensation prior to PKMK (54.7%) [27]. A similar rate of legal blindness was reported by Chen et al., with 67% of patients (n = 42) falling below the 20/200 threshold [28]. Furthermore, Harris et al. documented a decline in VA from a median of 20/200 to counting fingers at the last follow-up, with 15% of eyes loosing light perception entirely [29]. In a study of comparable sample size, Wagoner et al. found that only 21.6% of patients achieved a final VA of 20/200 or better, and just 8.2% achieved 20/40 or better [13]. Most studies to date have primarily focused on graft survival, consistently demonstrating that previously failed grafts are associated with the poorest outcomes, with a 5.6% survival rate at four years.

Medically controlled PKMK accounts for approximately 60–80% of cases [30]. When medical treatment fails, therapeutic keratoplasty is the next step, with graft survival achieved in approximately 80% of cases. However, the risk of eyeball removal (enucleation or evisceration) increases with subsequent graft failures and the development of concomitant endophthalmitis [31]. Optical grafts show a slightly higher success rate compared to therapeutic grafts but are still frequently complicated by loss of graft transparency over time [32]. According to the literature, the need for eyeball removal in PKMK cases varies from 0% to 21%, with the highest rate reported by Chatterjee et al., who studied PKMK exclusively in previous therapeutic grafts [25]. Consistent with other studies, nearly one-fourth of our cohort required emergency surgical intervention, either therapeutic full-thickness keratoplasty (18%) or evisceration (6.4%). Our findings align with the previously reported rates of eyeball removal: 5.8% in the study by Dohse et al. (n = 86 PKMK cases), 7.6% in the study by Lamas-Francis et al. (n = 654 eyes of culture-proven microbial keratitis), and 10% in the study by Valpayee et al. (n = 50) [10,33,34]. It should, however, be noted that the study by Valpayee et al. was conducted in a developing country between 1998 and 1999, during a period of limited understanding and evolving management of microbial keratitis.

There are several limitations to this study. This is a single-center study, which may limit the generalizability of the findings. Diagnostic and treatment patterns can vary across institutions, and geographical differences may also influence the incidence and clinical outcomes. Furthermore, the absence of a comparative control group limits the ability to distinguish factors specifically associated with infection risk from those related to overall keratoplasty prognosis. Due to the widespread geographic distribution of patients across the country, some individuals may have been lost to follow-up. However, severe and non-healing corneal ulcers are primarily referred to our tertiary center, which mitigates this concern to some extent. Another limitation is the relatively short history of posterior lamellar transplants at our institution, which may bias the observed rate of PKMK in DSAEK/DMEK patients. The retrospective nature of this study limited our ability to retrieve complete corneal culture results, particularly for the years 2008–2015, when only paper-based microbiological records were available. Furthermore, most patients were referred to our tertiary center with non-healing corneal ulcers that were already being treated empirically. As a result, corneal cultures were not routinely performed at the time of presentation due to the ongoing administration of broad-spectrum antimicrobial therapy. Finally, fungal keratitis in corneal transplant recipients may have a detrimental impact on visual outcomes. However, due to the low sensitivity of microbiological cultures for detecting fungal pathogens, and the fact that post-transplant keratitis patients typically receive multiple antimicrobial therapies, it is difficult to fully assess this impact in a retrospective study.

In conclusion, the rate of PKMK observed in our cohort (4.35% for the entire cohort, 5.9% in penetrating keratoplasties, and 0.37% for posterior lamellar grafts) aligns with the rates reported in most European and American studies. Among the studied risk factors, corneal perforation and systemic immunosuppression were found to significantly negatively impact the final visual outcome. Median BCVA decreased from 1.6 logMAR prior to PKMK to 2.3 logMAR at the time of infection, and improved to 1.9 logMAR at the one-year follow-up. A relative loss of vision was observed in 46% of patients who experienced graft infection. Notably, three-fourths of the cohort remained legally blind (BCVA > 20/200) one year following the infection episode. To the best of our knowledge, this represents one of the largest cohorts of corneal grafts screened for microbial keratitis in Europe. Additionally, for the first time, we assessed the role of general immunosuppression as a risk factor for post-keratoplasty microbial keratitis. Our findings can enhance patient counseling by providing insights into the risk of infection following corneal transplantation and identifying the key factors that may help to mitigate this risk. Furthermore, these results contribute to a deeper understanding of the potential course of infectious keratitis in transplanted corneas, offering valuable guidance for clinicians in their daily practice.

## Figures and Tables

**Table 1:** Demographics of the post-keratoplasty microbial keratitis patients, treated in 2008–2023 in the tertiary center.

Variable	Whole Cohort	Penetrating Keratoplasty (PKP)	Anterior Lamellar Keratoplasty (DALK)	Posterior Lamellar Keratoplasty (DSAEK)	*p* *
Number of transplants performed in our centre in 2008–2023, n (%)	2869 ^&^	1897 (66.3)	114 (4)	821 (28.6)	-
Number of microbial keratitis cases, n (%)	125 (100.0)	112 (89.6)	10 (8)	3 (2.4)	-
Age at microbial keratitis, mean ± SD	62.73 ± 17.45	63.21 ± 17.17	53.30 ± 19.73	76.00 ± 5.29	0.086 ^1^
Sex, female, n (%)	64 (51.2)	58 (51.8)	4 (40.0)	2 (66.7)	0.701 ^3^
Eye, right, n (%)	62 (50.4)	58 (52.7)	3 (30.0)	1 (33.3)	0.296 ^3^
Systemic autoimmunological disease, n (%)	41 (32.8)	39 (34.8)	1 (10.0)	1 (33.3)	0.163
Treatment mode, n (%)					
Intpatient	114 (93.4)	102 (93.6)	9 (90.0)	3 (100.0)	0.516
Outpatient	8 (6.6)	7 (6.4)	1 (10.0)	0 (0.0)	
Hospitalization, days, median (IQR)	9.00 (7.00; 12.00)	9.00 (7.00; 12.00)	3.00 (2.00; 12.00)	13.00 (10.50; 17.00)	0.200 ^2^

SD—standard deviation, IQR—interquartile range. Numeric variables compared with *t*-Student test ^1^ or Mann–Whitney U test ^2^. Nominal variables compared with Pearson’s chi-squared test ^3^ or Fisher’s exact test. * Comparison between PK and DALK. ^&^ 37 limbal transplants were excluded from the analysis (1.3%).

**Table 2:** Clinical history of corneal transplants.

Variable	Whole Cohort	Penetrating Keratoplasty (PK)	Anterior Lamellar Keratoplasty (DALK)	Posterior Lamellar Keratoplasty (DSAEK)	*p*
Number of microbial keratitis cases, n (%)	125 (100.0)	112 (100.0)	10 (100.0)	3 (100.0)	-
Recurrent infections, n (%)	72 (57.6)	67 (59.8)	4 (40.0)	1 (33.3)	0.317
Time from the last transplant to microbial keratitis, months, median (IQR)	17.00 (6.00; 40.00)	17.00 (5.75; 39.25)	18.50 (11.25; 57.50)	7.00 (6.00; 45.00)	0.611
Time from the first transplant to microbial keratitis, months, median (IQR)	76.00 (34.25; 143.75)	81.00 (35.00; 144.00)	12.00 # (12.00; 12.00)	-	0.169
Indication for transplantation, n (%)					
Active infection	65 (52.0)	59 (52.7)	6 (60.0)	0 (0.0)	0.196
Post-infection corneal scar	13 (10.4)	11 (9.8)	2 (20.0)	0 (0.0)	
Keratoconus	12 (9.6)	12 (10.7)	0 (0.0)	0 (0.0)	
Bullous keratopathy	22 (17.6)	19 (17.0)	0 (0.0)	3 (100.0)	
Immunological	3 (2.4)	2 (1.8)	1 (10.0)	0 (0.0)	
Chemical burn	5 (4.0)	5 (4.5)	0 (0.0)	0 (0.0)	
Corneal dystrophy	1 (0.8)	1 (0.9)	0 (0.0)	0 (0.0)	
Other	4 (3.2)	3 (2.7)	1 (10.0)	0 (0.0)	

# There is only 1 person in this group.

**Table 3:** Risk factors for post-keratoplasty microbial keratitis.

Variable	Whole Cohort	Penetrating Keratoplasty (PKP)	Anterior Lamellar Keratoplasty (DALK)	Posterior Lamellar Keratoplasty (DSAEK)	*p*
Contact lens use, n (%)	2 (1.8)	2 (2.0)	0 (0.0)	0 (0.0)	>0.999
Trauma, n (%)	3 (2.7)	3 (3.0)	0 (0.0)	0 (0.0)	>0.999
Glaucoma topical treatment, n (%)	62 (52.5)	55 (51.4)	4 (50.0)	3 (100.0)	>0.999
Glaucoma surgical history, n (%)	28 (23.9)	23 (21.7)	3 (37.5)	2 (66.7)	0.380
Broken/loose suture, n (%)	5 (4.0)	4 (3.6)	1 (10.0)	0 (0.0)	0.353
Dry eye syndrome, n (%)	41 (32.8)	37 (33.0)	3 (30.0)	1 (33.3)	>0.999
Recurrent erosions, n (%)	46 (36.8)	42 (37.5)	2 (20.0)	2 (66.7)	0.327
Local immunosuppression at the time of infection, n (%)	66 (56.4)	62 (58.5)	2 (25.0)	2 (66.7)	0.135
Systemic immunosuppression, n (%)	59 (50.4)	58 (54.7)	1 (12.5)	0 (0.0)	0.028
Systemic immunosuppression—type, n (%)					
MFM ^&^	18 (30.5)	18 (31.0)	0 (0.0)	0 (0.0)	0.305
Steroids	14 (23.7)	13 (22.4)	1 (100.0)	0 (0.0)	
MFM + steroids	23 (39.0)	23 (39.7)	0 (0.0)	0 (0.0)	
Others	4 (6.8)	4 (6.9)	0 (0.0)	0 (0.0)	
Previous immunosuppression, n (%)	59 (74.7)	52 (74.3)	4 (66.7)	3 (100.0)	0.651
Previous immunosuppression—type, n (%)					
Steroids	20 (45.5)	15 (39.5)	2 (66.7)	3 (100.0)	0.756
MFM	6 (13.6)	6 (15.8)	0 (0.0)	0 (0.0)	
MFM + steroids	18 (40.9)	17 (44.7)	1 (33.3)	0 (0.0)	
Time from ceased immunosuppression, months, median (IQR)	22.50 (8.50; 43.50)	22.50 (10.75; 36.00)	46.00 (23.50; 109.00)	4.00 (2.50; 37.00)	0.616
Active uveitis, n (%)	29 (23.2)	28 (25.0)	0 (0.0)	1 (33.3)	0.114
Therapeutic transplant, n (%)	19 (17.3)	18 (18.4)	0 (0.0)	1 (50.0)	0.208

MMF = mycophenolate mofetil.

**Table 4:** Vision-related clinical characteristics of the infectious keratitis in corneal transplant in the groups receiving systemic immunosuppression or not.

Variable	Whole Cohort	Penetrating on IMT	No IMT	*p*
BCVA—diagnosis, median (IQR)	2.30 (1.50; 2.30)	1.90 (1.23; 2.30)	2.30 (1.60; 2.70)	0.006 ^1^
BCVA—discharge, median (IQR)	1.90 (1.30; 2.30)	1.90 (1.30; 2.30)	2.30 (1.50; 2.40)	0.052 ^1^
BCVA—year one follow up visit, median (IQR)	1.90 (0.90; 2.30)	1.90 (0.70; 2.30)	1.90 (0.90; 2.40)	0.639 ^1^
BCVA—visit preceding the microbial keratitis onset, median (IQR)	1.60 (0.88; 2.30)	1.30 (0.80; 1.90)	1.60 (1.00; 2.30)	0.022 ^1^
Lens status, n (%)				
Phacic, transparent PCIOL ACIOL Cataract Aphacic	26 (22.6)	15 (26.3)	11 (19.3)	0.230
	65 (56.5)	33 (57.9)	32 (56.1)	
	0 (0.0)	0 (0.0)	0 (0.0)	
	13 (11.3)	7 (12.3)	6 (10.5)	
	11 (9.6)	2 (3.5)	8 (14.0)	
Decompensated graft prior to IK, n (%)	64 (54.7)	34 (57.6)	30 (51.7)	0.649
Recurrent erosions, n (%)	46 (36.8)	26 (44.1)	20 (34.5)	0.383
Dry eye syndrome, n (%)	41 (32.8)	20 (33.9)	21 (36.2)	0.946
Perforation, n (%)	16 (12.8)	6 (10.2)	9 (15.5)	0.556
Number of lesions, n (%)				
Multifocal Monofocal	15 (12.8)	7 (12.7)	8 (14.5)	>0.999
	102 (87.2)	48 (87.3)	47 (85.5)	
Therapeutic transplant, n (%)	19 (17.3)	6 (11.5)	11 (21.6)	0.269
Topical treatment, n (%)				
Monotherapy Politherapy	22 (18.0)	10 (16.9)	11 (19.6)	0.895
	0 (0.0)	0 (0.0)	0 (0.0)	
Evisceration, n (%)	10 (8.0)	6 (10.2)	4 (6.9)	0.762
Part of the cornea affected, n (%)				
Central Midperipheral Peripheral Whole cornea	61 (54.0)	29 (54.7)	29 (54.7)	0.736 ^2^
	24 (21.2)	9 (17.0)	13 (24.5)	
	23 (20.4)	12 (22.6)	9 (17.0)	
	5 (4.4)	3 (5.7)	2 (3.8)	

IQR—interquartile range. Numeric variables compared with *t*-Student test ^1^ or Mann–Whitney U test ^2^.

**Table 5:** Visual acuity parameters in the four studied timepoints.

Variable	Whole Cohort	Penetrating Keratoplasty (PKP)	Anterior Lamellar Keratoplasty (DALK)	Posterior Lamellar Keratoplasty (DSAEK)	*p*
BCVA—diagnosis, median (IQR)	2.30 (1.50; 2.30)	2.30 (1.52; 2.30)	1.30 (0.50; 2.30)	2.30 (2.10; 2.30)	0.094
BCVA—discharge, median (IQR)	1.90 (1.30; 2.30)	1.90 (1.30; 2.30)	1.75 (0.50; 2.40)	1.90 (1.90; 2.10)	0.550
BCVA—year one follow-up visit, median (IQR)	1.90 (0.90; 2.30)	1.90 (0.90; 2.30)	2.10 (1.43; 3.03)	1.40 (1.15; 1.65)	0.439
BCVA—visit preceding the microbial keratitis onset, median (IQR)	1.60 (0.88; 2.30)	1.60 (0.90; 2.30)	1.30 (1.15; 2.00)	1.30 (1.00; 1.30)	0.714

**Table 6:** Surgical interventions in the eyes with one and multiple corneal grafts.

Variable	One Corneal Transplant	Multiple Corneal Transplants (2–5 Grafts)	*p*
Therapeutic transplant, n (%)	11 (15.3)	7 (19.4)	0.784
Amniotic membrane, n (%)	17 (20.7)	7 (17.5)	0.858
Evisceration, n (%)	8 (9.8)	2 (5.0)	0.495 ^1^
Topical treatment, n (%)			
Monotherapy Politherapy	17 (21.0)	5 (12.8)	0.406
	64 (79.0)	34 (87.2)	
General treatment	47 (57.3)	22 (55.0)	0.962

Variables compared with Pearson’s chi-squared test or Fisher’s exact test ^1^.

**Table 7:** Outcomes of linear regression analysis for relative change in BCVA (the difference between BCVA at the visit preceding microbial keratitis onset and BCVA at the one-year follow-up visit).

Variable	Univariate Model				Multivariate Model			
	ß	95% CI for ß	Std. ß	*p*	ß	95% CI for ß	Std. ß	*p*
Age, years	0.00	−0.01 to 0.01	0.06	0.523	-	-	-	-
Sex, female (vs. male)	−0.31	−0.61 to −0.01	−0.39	0.041	-	-	-	-
Transplant type								
DALK (vs. PKP)	0.53	−0.06 to 1.11	0.65	0.077	0.65	0.09 to 1.21	0.83	0.024
DSAEK (vs. PKP)	0.16	−0.97 to 1.30	0.20	0.775	−0.30	−1.71 to 1.11	−0.38	0.677
Number of transplants	−0.05	−0.24 to 0.15	−0.05	0.633	-	-	-	-
Hospitalization, days	−0.02	−0.05 to 0.00	−0.16	0.109	-	-	-	-
Recurrent infections	0.32	0.01 to 0.63	0.40	0.040	-	-	-	-
Time from the last transplant to microbial keratitis, months	0.00	0.00 to 0.00	−0.11	0.265	-	-	-	-
Time from the first transplant to microbial keratitis, months	0.00	0.00 to 0.00	−0.32	0.072	-	-	-	-
Indication								
Post-infection corneal scar (vs. active infection)	0.01	−0.49 to 0.52	0.02	0.960	-	-	-	-
Keratoconus (vs. active infection)	−0.53	−1.05 to −0.01	−0.65	0.047	-	-	-	-
Bullous keratopathy (vs. active infection)	−0.31	−0.73 to 0.11	−0.39	0.141	-	-	-	-
Immunological (vs. active infection)	−0.76	−1.70 to 0.18	−0.94	0.110	-	-	-	-
Chemical burn (vs. active infection)	−0.39	−1.13 to 0.35	−0.48	0.299	-	-	-	-
Other (vs. active infection)	−0.35	−1.17 to 0.47	−0.44	0.394	-	-	-	-
CL use	−0.05	−1.21 to 1.11	−0.07	0.927	-	-	-	-
Trauma	−0.21	−1.16 to 0.74	−0.26	0.664	-	-	-	-
Dry eye syndrome	−0.13	−0.45 to 0.19	−0.16	0.426	-	-	-	-
Recurrent erosions	0.04	−0.27 to 0.35	0.05	0.785	-	-	-	-
Perforation	0.63	0.20 to 1.06	0.78	0.005	0.77	0.36 to 1.17	0.98	<0.001
Local immunosuppression at the time of infection	−0.24	−0.54 to 0.06	−0.29	0.123	-	-	-	-
Systematic immunosuppression	0.25	−0.04 to 0.55	0.32	0.094	0.45	0.16 to 0.74	0.57	0.003
Systematic immunosuppression—type								
Steroids (vs. MFM)	−0.06	−0.69 to 0.56	−0.07	0.843	-	-	-	-
MFM + steroids (vs. MFM)	−0.04	−0.58 to 0.50	−0.05	0.873	-	-	-	-
Others (vs. MFM)	0.09	−0.98 to 1.17	0.11	0.860	-	-	-	-
Previous immunosuppression	0.10	−0.28 to 0.49	0.14	0.591	-	-	-	-
Time from ceased immunosuppression, months	0.00	−0.01 to 0.00	−0.09	0.619	-	-	-	-
Active uveitis	0.18	−0.18 to 0.53	0.22	0.332	-	-	-	-
Therapeutic transplant	−0.38	−0.79 to 0.03	−0.49	0.067	-	-	-	-
BCVA—diagnosis	0.13	−0.11 to 0.36	0.11	0.282	-	-	-	-
Topical treatment, monotherapy (vs. polytherapy)	−0.13	−0.52 to 0.26	−0.16	0.522	-	-	-	-

ß—beta coefficient, CI—confidence interval, and std. ß—standardized beta coefficient.

## Data Availability

The data supporting the findings of this study are available from the corresponding author upon reasonable request.
